# How do EFL learners process oral tasks with different complexity: an exploratory study

**DOI:** 10.3389/fpsyg.2023.1241964

**Published:** 2023-11-02

**Authors:** Jiaxin Xing

**Affiliations:** College of Foreign Languages, Qufu Normal University, Qufu, China

**Keywords:** task complexity, oral production, oral performance, cognitive processes, EFL learners

## Abstract

The effects of task complexity on learners’ performance has been a much-researched issue in SLA field. However, until now many studies fail to provide empirical evidence of the effects of task complexity on learners’ processing. To fill the gap, this study examined how task complexity affects L2 learners’ cognitive processes with reference to Levelt’s speech production model (1989). Ten Chinese EFL learners were asked to complete two narrative tasks with different complexity manipulated by +/− few elements under the same planning conditions. Results revealed that: (1) in the complex task learners showed a slightly lower percentage of cognitive processes at the stage of conceptualization and formulation and a higher percentage linked to monitoring at the stage of comprehension; (2) learners’ fluency in oral performance was affected by the cognitive processes at all the stages of oral production. Accuracy seemed to be most enhanced by learners’ form monitoring at the comprehension stage. The study sheds light on how learners process the tasks with different complexity when producing the language, and is of implication to task-based language teaching.

## 1. Introduction

With the rise and development of task-based language teaching, task has become a basic unit in second language teaching, and the word “task” has also become one of the high-frequency words in the field of second language research. The enthusiasm of researchers for task research is largely due to the fact that tasks provide context for second language use and acquisition (Kim, 2009). As one of the important characteristics of tasks, task complexity has become the focus of second language task research. Studies on task complexity roughly fall into three categories: (1) task complexity and learner interaction (e.g., Nuevo, 2006; Robinson, 2007; Kim, 2009); (2) task complexity and learner performance (e.g., Skehan, 2014; Wang et al., 2020; Zheng and Liu, 2020; Chen, 2022; Xu et al., 2022; Xu and Zhang, 2023); and (3) task complexity and language acquisition/development (e.g., Révész, 2009, 2011). Among the above, the impact of task complexity on learners’ performance has attracted the most attention. However, the findings of the exact effects of task complexity are still not clear, and even sometimes are inconsistent. In addition, despite much research investigating the relationship between task complexity and task performance, there is a surprising lack of research to gain insights into learners’ cognitive processes when carrying out oral tasks (Révész, 2014; Tao and Wang, 2019). Therefore, further research is warranted.

## 2. Literature review

In the field of task-based research, the Limited Attention Capacity Hypothesis (LACH) (Skehan, 1996, 2003, 2014) and the Cognition Hypothesis (CH) (Robinson, 2001, 2005, 2011) are the most influential and have inspired many studies (Norris et al., 2014). On the basis of LACH, it is argued that a trade-off may exist between attention to form and attention to meaning during task performance. As a result, more complex tasks will demand more attention to content, and correspondingly will allow less attention to form (Skehan, 1998; Skehan and Foster, 1999, 2001).

Robinson (2001) defined task complexity as “the attention, memory, reasoning, and other information processing loads caused by task structure to learners” (p. 29), and distinguished resource-directing and resource-dispersing task complexity. The former mainly includes the number of elements involved in the task, spatio-temporal characteristics and reasoning needs, while the latter mainly refers to whether learners have time to plan, and how much previous relevant knowledge learners have. According to Robinson, there is not a trade-off between attention to accuracy and attention to the complexity of language production. Rather, he claims that making tasks more complex in resource-directing dimensions will increase linguistic accuracy and complexity simultaneously. So far, researchers have done a lot of research on task complexity. Among them, the relationship between task complexity and learners’ language production has attracted much attention (Ishikawa, 2008; Robinson, 2011; Skehan, 2014; Wang et al., 2020). However, the conclusions on how task complexity affects learners’ attention allocation, and then how it affects form and meaning are not consistent (Xing and Luo, 2016).

Although a large number of studies have examined learners’ task performance, few have focused on learners’ second language production process. Ma (2013) found that learners focus more attention resources on speech processing in different oral tasks and less on the process of speech monitoring. The processing and monitoring of speech forms are more than the processing and monitoring of speech content. Although this research explored learners’ cognitive processes, it explored the effects of task types rather than task complexity. Recently, more and more scholars began to investigate the impact of task complexity on learners’ processing. For example, Révész et al. (2017) examined the impact of task complexity on the cognitive behavior of 4 s language learners. The results showed that content support might reduce the processing burden of learners’ conceptualizing process, promote the attention to language coding, and help improve lexical complexity. However, this study focused on writing rather than oral speaking. Xu and Chen (2018) examined the impact of task complexity on learners’ oral performance and attention allocation. It was found that with the increase of task complexity, learners paid more attention to language forms. Although this study focused on oral production, the annotation and classification of stimulated recall were relatively broad, lacking detailed analysis of learners’ cognitive activities, and involving a small sample.

In general, the existing studies in the field of second language tasks are basically aimed at investigating the impact of task complexity on learners’ language performance, while the impact of task complexity on learners’ processing is largely under-researched (Pang and Skehan, 2014; Révész, 2014; Révész et al., 2017). More research is needed for in-depth and detailed discussion. In view of this, the present study intends to explore the impact of task complexity on learners’ cognitive process of oral production.

## 3. Methodology

### 3.1. Research questions

This study aims to answer the following questions:

1. How does task complexity affect the proportion of cognitive activities at different stages of learners’ oral production?
2. What are the characteristics of learners’ cognitive activities when they complete tasks with different complexity? How are the cognitive activities related to oral performance?

### 3.2. Participants

The paiticipants of this study were 10 first-year non-English majors from a university in Eastern China. All of them were native Chinese speakers and had learned English for more than 9 years. They were non-English majors, but English was an obligatory course. None of them had been to English-speaking countries at the time of the study. All the subjects volunteered to participate in this study.

### 3.3. Research design

The study was designed as a repeated measurement experiment within the subject. The participants were randomly divided into two groups. The first group completed simple tasks first, and then completed the corresponding complex tasks. The second group completed oral tasks in reverse order. In order to minimize the repetition effect between tasks, the second time was finished 2 weeks later. All the participants completed the oral task independently in turn, and the time for each subject to produce the task and stimulate recall was about 30 min each time.

### 3.4. Research instruments

#### 3.4.1. Oral tasks

Two oral tasks were designed with different task complexity were designed for the participants. Consistent with relevant research in the field of second language task research (e.g., Michel et al., 2007; Michel, 2013), this study controlled task complexity based on the +/− few elements involved in the task, which was defined as whether the pictures described by the subjects concerned more or fewer elements. In the complex task, the researcher presented the subjects with a group of pictures that involved more elements. In the simple task, the subjects described a group of pictures involving fewer elements. To finish the task, the subjects needed to make a decision on the best matches of dating according to the personal information provided by the pictures. In the simple task, there were two men and two women, a total of four contestants for the subjects to choose, and in the complex task, there were three men and three women. Since the complex task includes more elements, it was believed to impose higher cognitive demand on the participants (Robinson, 2007). The pictures used in the task were determined after the pilot study.

#### 3.4.2. Stimulated recall

Relevant studies have proved the effectiveness of using stimulated recall to explore the psychological mechanisms of second language learners (such as Polio et al., 2006; Fu and Li, 2017). In order to explore the cognitive process of the subjects as accurately as possible, the stimulus recall was conducted immediately after each task was completed. While watching their own videos, the subjects recalled the psychological process when completing the task. When the subjects recalled some activities, they asked the researcher to pause the video and told what they thought at that time. In the process of watching the video, the researcher also paid attention to the nonverbal factors, and paused the video to ask about the subjects’ thoughts when necessary. The subjects recalled in their mother tongue, and the researcher recorded the entire process of the subjects’ recall.

### 3.5. Data collection

The data in this study was mainly from oral task production recordings and stimulated recall recordings. In order to avoid interference from external factors, the recordings were conducted in a quiet place. The researcher recorded the subjects’ oral production with an MP3 recorder, recorded the video with a QuickTime player, and transcribed the audio files with Soundscriber. All the recording files were transcribed by English major postgraduates, and the researcher proofread the transcribed texts one by one. After transcribing, a total of 20 oral task recording texts and 20 stimulated recall texts were obtained. All the texts were used for further analysis to answer the two research questions.

The researchers also conducted classification and tagging on the recorded texts of stimulated recall. The identification and classification of cognitive activities are based on the speech production model of Levelt (1989). First, according to the time of subjects’ cognitive activities, they were divided into two categories: pre-articulation and post-articulation. If what the participants recalled is about one or more utterances that have not been uttered, it is categorized as a pre-articulation process. If the recall is about what has already been uttered, it is classified as the post-articulation processes. Then, the cognitive activities were classified as about content or form. After determining the content or form, it can be subdivided according to specific characteristics (Ma, 2013). Pre-articulation processes related to content are considered to fall into the conceptualization phase in Levelt’s model; processes about linguistic forms are grouped into the formulation phase. Post-articulation processes draw strongly on the comprehension system. In addition, the processes are divided into processing and monitoring (see Ma, 2013). Processing includes content processing and form processing. Similarly, monitoring is made up of content monitoring and form monitoring.

With regard to monitoring, according to Levelt’s model, the speaker carries out three rounds of monitoring. The first round checks whether the message is consistent with the speakers’ original purpose; the second takes place before the articulation and checks the linguistic forms; the last checks the speech content and form after articulation. Since the first round of monitoring is about the content to express, such instances were categorized into the conceptualization stage. The second round was assigned to the formulation stage, for it is related to linguistic forms before articulation. The last round of monitoring falls into the comprehension stage since it happens after articulation. This method of categorization is also in line with some studies exploring processes of learners’ speech behaviour (e.g., Ma, 2013). The general coding scheme is shown in Table 1.

**Table 1:** General coding scheme of participants’ cognitive processes.

Pre-articulation	Conceptualization	Content processing Content monitoring (round 1)
	Formulation	Form processing Form monitoring (round 2)
Post-articulation	Comprehension	Content monitoring (round 3) Form monitoring (round 3)

In order to ensure the reliability of the statistical results, the researcher invited another English teacher to code, classify and calculate 10 randomly selected stimulated recall texts based on Levelt’s framework. After the independent annotation, the researcher and the teacher compared the annotations and discussed about the differences until they were completely consistent. Finally, the researcher annotated and counted all other stimulated recall texts.

The following is an example of the annotation of the stimulated recall text of the subjects in this study:

*Content organization:* The … um the first reason is … the age uh … James is 22 years old. Mary is 23 years old.

(Recall: *I wanted to say their ages are close, but I was afraid that I could not speak a lot. So I decided to tell their ages one by one so that I could say more.) (note: all the transcriptions of stimulated recall were translated into English*.)

*Choice of words:* And they both like rock they both like sporting sports.

(Recall: *I was not sure whether I should use sporting or sports*.)

*Content searching:* Peter uh like like metal music metal music, Susan like rock music. … …

(Recall: *I did not know what else to say. I felt that I have said a lot, so I was thinking about what to say.*)

### 3.6. Data analysis

To answer the research questions, both quantitative and qualitative analysis were used. For the first research question, the researcher classified the cognitive activities of the participants according to the speech production model of Levelt, calculated the number of specific types of cognitive activities of the subjects, and conducted Chi-square test. All quantitative statistical analyses were performed using SPSS 20.0. For the second research question, the researcher made a detailed qualitative analysis of the characteristics of the types of learners’ cognitive activities by referring to the participants’ performance.

## 4. Results

### 4.1. The influence of task complexity on the participants’ cognitive activities

To answer the first question, we compared the proportion of cognitive activities at different stages of oral production in the simple task and the complex task. Referring to the framework of Levelt’s speech production model, 12 types of cognitive activities were obtained. The number of specific cognitive activities of subjects in simple tasks and complex tasks was investigated, respectively. Table 2 summarizes the distribution of specific cognitive activities of subjects in the two tasks.

**Table 2:** Stimulated-recall results: the incidence of the cognitive processes of 10 participants.

Category			Number/percentage	
Level 1	Level 2	Level 3	Simple	Complex
Conceptualization	Content processing	Content searching	11/19.64%	6/9.52%
		Content organization	3/5.36%	6/9.52%
	Content monitoring	Content accuracy	1/1.79%	0
Sub-total			**15/26.79%**	**12/19.05%**
Formulation	Form processing	Choice of expressions	16/28.57%	9/14.29%
		Choice of words	14/25%	21/33.33%
		Choice of grammar	1/1.79%	0
	Form monitoring	Lexical accuracy	0	4/6.35
		Lexical appropriacy	0	1/1.59
		Grammatical accuracy	3/5.36%	3/4.76%
Sub-total			**34/60.71%**	**38/60.32%**
Comprehension	Content monitoring	Content accuracy	1/1.79%	0
		Content appropriacy	0	2/3.17%
	Form monitoring	Lexical accuracy	2/3.57%	1/1.59%
		Lexical appropriacy	1/1.79%	0
		Grammatical accuracy	3/5.36%	10/15.83
Sub-total			**7/12.5%**	**13/20.63%**
Total			56	63

**Figure 1:**
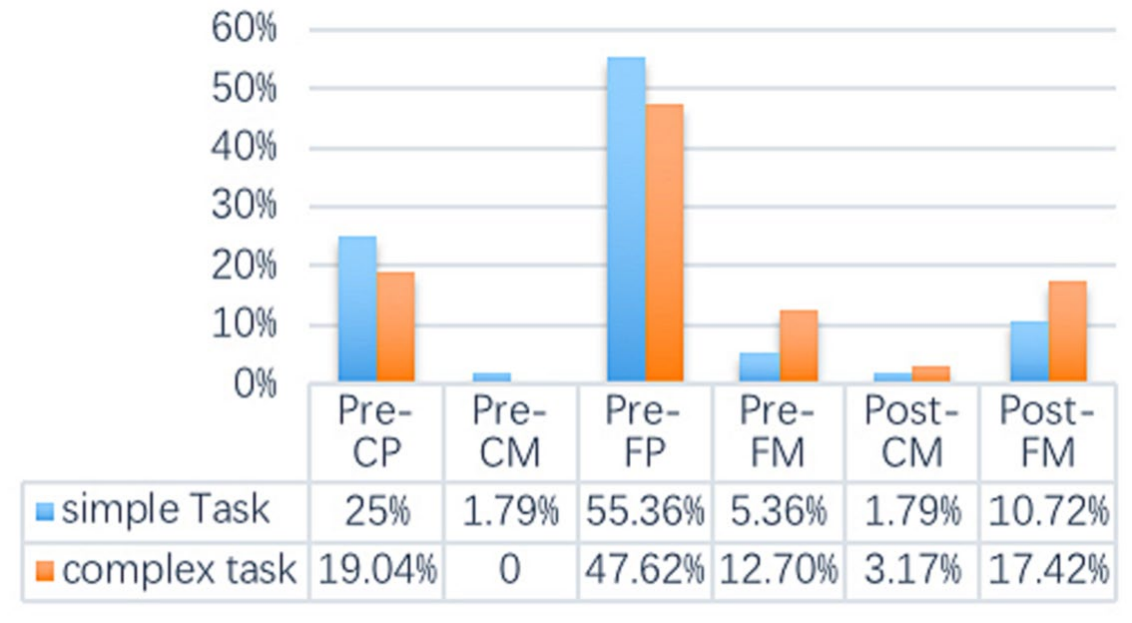
The distribution of the subjects’ cognitive Processes. CP, content processing; CM, content monitoring; FP, form processing; FM, form monitoring; Pre, pre-articulation; Post, post-articulation.

As is shown in Table 2, the distribution of cognitive activities of subjects in simple tasks and complex tasks is different to some extent. The proportion of subjects’ cognitive activities about content at the conceptualization stage of complex task is slightly lower, the number of cognitive activities at the formulation stage is slightly lower, but the proportion is higher at the comprehension stage. In complex tasks, the proportion of cognitive activities related to choice of expressions decreased significantly, while the proportion of activities related to choice of words increased slightly. At the three stages, the cognitive activities related to the comprehension stage are the most different, with the monitoring of grammatical accuracy most obvious (15.83% > 5.36%). In other words, the participants tend to demonstrate more monitoring of the accuracy of words in the complex task. According to the different mechanisms of articulation, processing and monitoring, 12 cognitive activities can be generalized into six categories. Figure 1 shows the distribution proportion of these six types of activities.

Figure 1 shows that the highest proportion of cognitive activities of the subjects in task production is pre-articulation form processing, which accounts for about half of the total in both simple and complex tasks (55.36% & 47.62%). The subjects’ monitoring of language forms after articulation also accounted for a relatively high proportion, especially in complex tasks, which accounted for nearly 20%. The differences in the distribution of cognitive activities of the subjects in the production of simple tasks and complex tasks were shown in almost all six types of activities. Among them, the most obvious difference lies in the feature of pre-articulation form processing and post-articulation form monitoring. The cognitive activities of the subjects on post-articulation form monitoring in complex tasks were about twice that in simple tasks.

The researchers used inferential statistics to further investigate the differences in the distribution of cognitive activities in the output of simple tasks and complex tasks. Chi-square test shows that there is no significant difference in the overall cognitive activity distribution at the three stages of speech production (*χ*2 =1.951, df = 2, *p* = 0.377), and the difference of the distribution of choice of expressions and choice of words is close to the significant level (*χ*2 =3.360, df = 1, *p* = 0.067). Results indicate that participants’ cognitive activities in the simple task and the complex task were not significantly different.

### 4.2. The characteristics of learners’ cognitive activities in the simple task and the complex task

#### 4.2.1. Cognitive activities at the conceptualization stage

As is shown in Table 2, the percentage of cognitive activities in the simple task was slightly higher than that in the complex task. Closer examination showed that whether the participants searched for the content or organized the content to be expressed, they usually had a long pause or a large number of repeated words, thus reducing the fluency of oral production. The monitoring of content accuracy of the subjects usually affected their oral fluency. These findings can be shown in the following examples in the simple task.

1. And and I think they all are and they all quiet. (ST01)
(Recall: *Here I was thinking about their similarities. One likes reading books, the other likes watching movies. These are both hobbies which are comparatively quiet, so I was thinking about how to express that.*)
2. … … uh and Mary read many read many books, Peter read newspaper. (ST03)
(Recall: *I paused here because I was thinking about that to say next.*)
3. because they no smoking and uh … and … and … sport sport sometimes. (ST08)
(Recall: *I was thinking about what else makes them not suitable for each other. I tried to work out what to say next.*)

#### 4.2.2. Cognitive activities at the formulation stage

At the formulation stage, the cognitive activities about form processing in the two task productions were almost the same. Specifically, the percentage related to choice of expressions in the complex task decreased, while the percentage related to vocabulary expression increased. These two types of activities constitute the main categories of cognitive activities of the task output, which usually reduce the fluency. This can be illustrated in an example in the complex task:

4. Uh … the music they like uh are all uh … … uh are all uh … … are the same uh … are the same type. (CT03)
(Recall: *I wanted to say what type of music they both like, but I could not find the proper adjectives. I just said they liked the same type.*)

In addition to the influence of fluency, learners’ choice of expression or words also had an impact on vocabulary use and language accuracy. As for the choice of words, the subjects were usually unable to find a corresponding target word. The strategies they often used were mainly replacement or to give up. For example, the following cases were found in the complex task:

5. They do not … uh they both … uh … the … they both like uh … many kinds of books. (CT04)
(Recall: *I wanted to say they were not picky about the type of books, but I did not know the word*.)
6. Um I will … I will tell you which which which partici um … which one I would like to choose. (CT05)
(Recall: *I did not know the word “participant,” then chose to use another which is easier to replace it.*)
7. They they all like um … … Susan like … … read books and Martin also like read books. (CT02)
(Recall: *I wanted to say they had similarity as for music, but I could not find an accurate word, so I finally described them one by one.*)

Example 5–7 show that when learners encountered difficulties in the choice of expression, one of the commonly used strategies was to use simple language form. Therefore, the increase of such cognitive processes did not generally improve learners’ lexical complexity. In addition, learners sometimes gave up the form of the target language and continued the production when they had difficulties in the choice of expression or words, or temporarily used the language form that they were uncertain about, which usually led to errors and then reduced the accuracy. Example 8 was from the simple task, and example 9 and 10 were from the complex task.

8. So I think Peter and Mary can have many same same … language. (ST06)
(Recall: *I wanted to say they had a lot in common, which made them easy to communicate. But I did not know how to say that then.*)
9. Um I think I think James and Mary uh is … I think James and Mary. (CT10)
(Recall: *I thought James and Mary were the most likely to make a pair, but I did not know how to say the word.*)
10.Uh … uh James can uh … … can help Susan uh … … get uh get up smoke. (CT03)
(Recall: *I was thinking about how to say “give up smoking.”*)

#### 4.2.3. Cognitive activities at the comprehension stage

In two oral tasks, the cognitive processes differed the most at the comprehension stage, especially in the proportion of formal monitoring. In terms of the proportion, the subjects’ monitoring of grammatical accuracy after articulation in the complex task was almost three times that in the simple task. Through the analysis of the characteristics of the grammatical monitoring of the subjects, it was found that the subjects’ grammatical monitoring was mostly due to the recognition of grammatical errors, so they tried to make corrections. This usually corrected the original grammatical errors, thus making the produced language conform to the grammatical norms. This might partly explain why the language in the complex task was more accurate than that in the simple task. The following examples (example 11 and 12 are from the simple task, example 13 and 14 are from the complex task) can illustrate this point well:

11. So I think they may be not stay at each other with each other finally. (ST02)
(Recall: *After I said that, I realized that I used the wrong preposition. So I corrected myself immediately.*)
12. And Peter like read like reading newspaper. (ST06)
(Recall: *I realized that like should be followed by -ing form.*)
13. but James uh … did not uh does not smoke. (CT03)
(Recall: *I used the wrong tense, so I corrected myself.*)
14. Susan um … Susan’s Susan … … like to smoking like to smoke. (CT05)
(Recall: *It should be “like to do,” not “doing.” So I corrected the mistake.*)

#### 4.2.4. A brief summary of the characteristics

From the above analysis, it could be seen that there was a certain relationship between the participants’ cognitive activities in the task production and their oral performance: (1) the participants’ language processing and language monitoring generally reduced fluency, and the most influential factors on fluency were content processing and form processing. (2) Pre-articulation form monitoring usually did not enhance the language accuracy, but it reduced the fluency. (3) The cognitive processes related to choice of expression or words were likely to reduce the language accuracy and lexical complexity. (4) The participants’ monitoring of language form at the comprehension stage made the language in complex tasks more accurate. Thus, the second research question was answered.

## 5. Discussion

### 5.1. The influence of task complexity on learners’ cognitive activities

The results of this study show that the distribution of learners’ cognitive activities in the complex task and simple task has both similarities and differences. The commonness lies in that most of the learners’ cognitive activities in the two tasks are related to form processing. The difference is reflected in the change of the specific proportion of the learners’ form processing in complex tasks, as well as more monitoring of language forms. The findings of this study on the proportion of overall cognitive activities of learners in the two tasks are consistent with Ma (2013) and Fu and Li (2017). The reason why learners’ large amount of cognitive activities in oral production are related to language form processing may be that second language learners often encounter difficulties in finding appropriate language forms (Ellis and Shintani, 2014). It may also be influenced by learning experience. English teaching accepted by the subjects in the foreign language environment in China is more grammar-centered, and more emphasis is placed on the accurate use of language forms (Xu and Chen, 2018), which makes learners pay too much attention to the accuracy of language when producing oral English.

This study also found that with the increase of task complexity, the proportion of learners’ cognitive activities related to speech monitoring increased (Table 2), which was different from Ma (2013) results. This may be because: firstly, the two studies employ different ways of manipulating task complexity. Our study controls task complexity based on +/− few elements rather than task types. Secondly, our study the subjects are non-English major undergraduates with relatively low English proficiency, while Ma (2013)‘s subjects are English major postgraduates with relatively high English proficiency. According to the speech production frameworks such as Levelt (1989); Kormos (2006), lower level learners will need more time to code their communicative intentions. All these will affect the specific process of language production. Xu and Chen (2018) also found that with the increase of task demands, learners paid more attention to language forms.

The processing and monitoring of content and form by learners can be explained by the limitation of attention resources. That is, the more attention resources learners use for content processing and form processing, the less attention resources they use for form monitoring. In the simple task, the subjects had more cognitive activities of pre-pronunciation content and form processing, and less cognitive activities of post pronunciation form monitoring. However, in the corresponding complex task, the activities related to processing are reduced, while the activities related to monitoring are increased. Perhaps it is precisely because learners allocate less attention resources to processing (including content and form) that they can conduct more formal monitoring. Ahmadian et al. (2012) supplemented the competition hypothesis based on their findings about learners’ self-correcting behavior. They believe that in addition to the competition between the complexity, accuracy and fluency of second language production, there is also a competition between the three rounds of monitoring of second language learners’ own speech. From the results of this study, there may also be a competitive relationship between learners’ processing and monitoring of content and form.

### 5.2. The relationship between learners’ cognitive processes and their oral performance

Through the analysis of the characteristics of the cognitive activities of the subjects, it is found that the complexity of the task affects the allocation of attention resources at different speech production stages, and ultimately affects the learners’ oral performance. In general, learners’ language processing or monitoring will reduce their oral fluency; The pre-articulation content and form processing of subjects have the greatest impact on oral fluency. Form processing usually reduces language accuracy, while post-articulation form monitoring is of great help to language accuracy (Lynch, 2007; Li, 2014).

Skehan (2009, 2011) made a detailed analysis of the relationship between task demand and speech production stages. He believes that the conceptualization stage of speech is most related to the complexity of language production, while the formulation stage is related to accuracy and fluency. According to Skehan, the impact of task characteristics on spoken language production is mainly due to their different efforts made by learners at the conceptualization stage and the lexical and syntactic encoding and formulation stage of speech production. This research supports Skehan’s view on the distribution proportion of pre-articulation content processing, pre-articulation form processing and pre-articulation form monitoring features. However, this study also finds that the influence of task complexity on learners’ oral production exists not only at the conceptualization stage and the formal stage, but also at the form monitoring efforts in the comprehension stage, which further enriches Skehan’s interpretation.

This study found that learners’ form monitoring at the stage of comprehension could promote language accuracy, which indicates the important role of language monitoring. Wang (2014) also believes that the quality of language monitoring is directly related to oral performance, especially language accuracy. The research of Hulstijn and Hulstijn (1984) can also provide us with inspiration. They found that when learners are guided to pay more attention to language forms in language production, language accuracy will be improved. This study failed to find the relationship between cognitive activities and language complexity, which may be because stimulated recall is more about the investigation of learners’ psychological activities when they are not fluent, and the influence of task conceptualization demands on syntactic complexity exists in fluent production to a large extent (Révész et al., 2017).

## 6. Conclusion

This study examined the effect of task complexity on learners’ cognitive process of oral production. It was found that task complexity had no significant impact on the overall proportion of cognitive activities at three stages of learners’ speech production. Despite that, the distributions of cognitive activities in the simple task and the complex task are not the same. Specifically, post-articulation monitoring of learners’ language forms usually improved speech accuracy. Based on the results of this study and previous studies, we suggest that teachers should give some guidance to learners before implementing tasks, and actively adopt various ways to improve learners’ awareness of language monitoring, so as to achieve the goal of focusing-on-Form.

The findings of this study are not conclusive especially due to the small sample size. Therefore, future research can include a larger sample to investigate learners’ oral task production process, and try to use eye movement, ERP and other research methods to better understand how task complexity affects learners’ cognitive processes.

## Data availability statement

The raw data supporting the conclusions of this article will be made available by the authors, without undue reservation.

## Ethics statement

Ethical review and approval was not required for the study on human participants in accordance with the local legislation and institutional requirements. The participants provided their written informed consent to participate in this study.

## Author contributions

The author confirms being the sole contributor of this work and has approved it for publication.
